# Prevalence of Nasopharyngeal Carcinoma in Patients with Dermatomyositis: A Systematic Review and Meta-Analysis

**DOI:** 10.3390/cancers13081886

**Published:** 2021-04-14

**Authors:** Ahmad Adebayo Irekeola, Rafidah Hanim Shueb, Engku Nur Syafirah E. A. R., Yusuf Wada, Zaidah Abdul Rahman, Suhana Ahmad, Rohimah Mohamud, Norhafiza Mat Lazim, Chan Yean Yean

**Affiliations:** 1 Department of Medical Microbiology and Parasitology, School of Medical Sciences, Health Campus, Universiti Sains Malaysia, Kubang Kerian 16150, Kelantan, Malaysia; profahmad007@yahoo.com (A.A.I.); engkunursyafirah@student.usm.my (E.N.S.E.A.R.); wadayusuf@student.usm.my (Y.W.); drzaidah@usm.my (Z.A.R.); yychan@usm.my (C.Y.Y.); 2 Microbiology Unit, Department of Biological Sciences, College of Natural and Applied Sciences, Summit University Offa, Offa PMB 4412, Nigeria; 3 Institute for Research in Molecular Medicine (INFORMM), Universiti Sains Malaysia, Kubang Kerian 16150, Kelantan, Malaysia; 4 Department of Zoology, Faculty of Life Sciences, Ahmadu Bello University, Zaria 810211, Nigeria; 5 Hospital Universiti Sains Malaysia, Universiti Sains Malaysia, Kubang Kerian 16150, Kelantan, Malaysia; 6 Department of Immunology, School of Medical Sciences, Health Campus, Universiti Sains Malaysia, Kubang Kerian 16150, Kelantan, Malaysia; suhanaahmad@usm.my (S.A.); rohimahm@usm.my (R.M.); 7 Department of Otorhinolaryngology-Head and Neck Surgery, School of Medical Sciences, Health Campus, Universiti Sains Malaysia, Kubang Kerian 16150, Kelantan, Malaysia; norhafiza@usm.my

**Keywords:** nasopharyngeal carcinoma, nasopharyngeal neoplasm, NPC, dermatomyositis, dermatopolymyositis

## Abstract

**Simple Summary:**

This first systematic review and meta-analysis on the prevalence of nasopharyngeal carcinoma in patients suffering dermatomyositis was necessitated by the absence of a true and reliable prevalence estimate necessary to adequately inform medical preparedness and decisions. Following a careful review of literature and data analyses, a prevalence of 3.3% was found. It is hoped that a clear knowledge of the actual prevalence of nasopharyngeal carcinoma in dermatomyositis patients would not only help sensitize clinicians and patients about the frequency of these disease conditions but would also enhance the adoption of precautions essential to mitigate their co-occurrence in patients.

**Abstract:**

For more than 50 years, nasopharyngeal carcinoma (NPC) has been associated with dermatomyositis (DM), a rare idiopathic inflammatory disorder that mainly affects the skin and muscles. Although the association between these rare diseases is well-documented, the actual prevalence of NPC in DM patients remains unknown. Here, a systematic review and meta-analysis of published data was conducted in accordance with the guidelines of Preferred Reporting Items for Systematic Reviews and Meta-Analysis (PRISMA). Electronic databases including PubMed, Scopus, ScienceDirect, and Google Scholar were searched without year or language restrictions for studies reporting the occurrence of NPC in DM patients. The study protocol was lodged with PROSPERO (CRD42021225335). A total of 95 studies covering 303 cases of NPC among 16,010 DM patients was included. Summary estimates were calculated using the random-effects model. The pooled prevalence of NPC in DM was 3.3% (95% CI, 2.5–4.3). When stratified according to study location, higher prevalence estimates were obtained for Hong Kong (36.5%), Malaysia (27.7%), and Singapore (11.9%). There was a predominance of cases among male DM patients compared with females, and most patients were aged 40 and above. Many of the NPC cases were found to be diagnosed after the diagnosis of DM. It is therefore pertinent to screen for NPC in DM patients, especially among older DM patients in the Asian region.

## 1. Introduction

Nasopharyngeal carcinoma (NPC) is a rare, malignant, non-lymphomatous, squamous-cell carcinoma arising from the epithelial lining of the nasopharynx [1,2]. The neoplasm is highly associated with latent infection of Epstein-Barr virus (EBV), an organism present in about 95% of the total population [2,3]. Although the occurrence of this type of tumor among patients had been documented prior to 1901 [4], one of the early most exhaustive studies describing the clinicopathological characteristics of NPC drawn from 114 patients in Hong Kong was published in 1941 [5]. Many reports involving a large number of NPC patients have now been recorded. Although the disease is infrequent in many countries, it poses a major health challenge in Southeast Asia, Southern China, the Arctic, North Africa and the Middle East [6].

Nonspecific epistaxis, audiologic symptoms, unilateral nasal obstruction to cranial nerve palsies and nodal metastasis in the neck region often characterize the clinical presentation of NPC [7,8]. To date, the precise cause of the disease remains unclear. However, an array of risk factors has been described including ethnicity, hereditary trends, dietary habits, tobacco smoking and infection with EBV [2,9].

The association of NPC with paraneoplastic syndromes is well established [10]. In this review, we focus on dermatomyositis (DM) due to the increasing reports of identified cases. DM is an uncommon idiopathic inflammatory disorder principally affecting the skin and muscles [11]. Its pathophysiology is not well understood. Patients typically present with cutaneous disease accompanied or briefly followed by proximal muscle weakness [11]. The seminal study reporting the association of NPC with DM was published in 1969 [12]. Since then, patients with DM have been closely observed for potential underlying malignancies, and a myriad of case reports and cases series have been described in the literature. NPC can precede, follow or be concurrent to the diagnosis of DM, indicating that one disease condition could be a risk for the other. Further, it is tempting to surmise that NPC patients have a higher tendency to develop DM.

It has been suggested that patients with malignancy-associated myopathies are more resistant to treatment compared with those without malignancy [13,14]. An unfavorable prognosis may also ensue when DM is associated with NPC. Many researchers thus recommend that patients with DM be screened for NPC [15,16,17,18]. Inarguably, an understanding of the prevalence of NPC in DM would help inform medical decisions. Although several isolated reports of the occurrence of NPC in DM patients abound, the true prevalence is yet unknown. Here, we present a first report of the actual prevalence of NPC in DM by pooling available published data using a meta-analytical approach.

## 2. Materials and Methods

A systematic review and meta-analysis of published articles was conducted according to the guidelines of the Preferred Reporting Items for Systematic Reviews and Meta-Analyses (PRISMA) [19]. The study protocol for this review was registered with PROSPERO (registration number: CRD42021225335).

### 2.1. Literature Search and Selection Criteria

**Figure 1:**
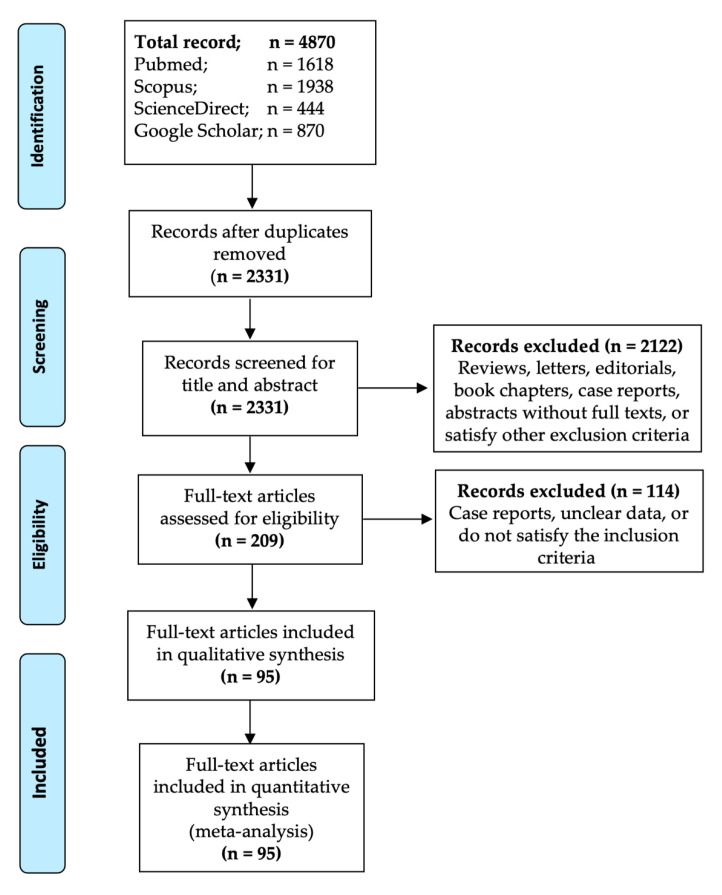
Summary of article identification and selection process.

Four electronic databases (PubMed, Scopus, ScienceDirect, and Google Scholar) were searched using a combination of relevant keywords: “nasopharyngeal carcinoma”, “NPC”, “nasopharyngeal neoplasm”, “nasopharyngeal cancer”, “malignancy”, “cancer”, “dermatomyositis”, “dermatopolymyositis” and “myositis”. Full details of the search strategies employed for all the searched databases are available as a Supplementary Document (File S1). The search was elaborate: filter for language, country, study design or year of publication was not applied. The initial search was done on 9 December 2020. The updated and final search returning a total of 4870 articles (Figure 1) was conducted on 11 March 2021. All references were exported to EndNote X8 software and were followed by duplicate removal.

Studies that investigated the occurrence of NPC and/or other cancers in patients suffering DM were considered for inclusion. We excluded (1) opinions, reviews, letters, book chapters, editorials and case reports; (2) studies that reported the occurrence of NPC in conditions other than DM; (3) studies whose NPC screening status were unclear or that did not include NPC among the cancers examined in DM patients and (4) articles whose full text could not be accessed. To ensure an exhaustive search, we perused and reviewed the references of the included studies.

All authors participated in delineating the article screening, selection, and assessment criteria. Two authors (A.A.I. and E.N.S.E.A.R.) independently screened the articles based on title and abstract. This was followed by the assessment of the full texts. Disagreements in the screening process were resolved by discussion including other authors.

### 2.2. Data Extraction and Quality Assessment

Data extraction was done using a predefined Excel spreadsheet. Three authors (A.A.I., S.A. and E.N.S.E.A.R.) independently extracted information regarding the study ID, year of publication, study period, study design, study location, number of patients involved, including their age and sex, number of NPC cases reported as well as the age and sex of the patients involved, and the period NPC was diagnosed among the cases.

The methodological quality of the included studies was assessed independently by two authors (A.A.I. and Y.W.) using the Joanna Briggs Institute (JBI) critical appraisal checklist for prevalence data [20] (File S2). A score of 1 for “yes” and 0 for other parameters were assigned to attain a total quality score that ranged from 0 to 9. Studies with an overall score of 7–9 were considered to be of sufficient quality.

### 2.3. Data Synthesis and Analysis

Data analysis was done using OpenMeta Analyst and meta (version 4.15-1) and metafor (version 2.4-0) packages of R (version 4.0.3) and RStudio (version 1.3.1093) [21]. The pooled prevalence of NPC in patients with DM was calculated, and subgroup analysis was done according to the location, geographical region and period of study. Random-effect model using the DerSimonian-Laird method of meta-analysis was used to derive the pooled estimates of the reported NPC cases. In addition to assessing study quality, potential publication bias was investigated by generating a funnel plot. The asymmetry of the plot was further assessed using Egger’s regression test [22]. The heterogeneities of study-level estimates were evaluated by Cochran’s Q test and quantified using *I*^2^ statistics. *I*^2^ values of 25%, 50%, and 75% were considered low, moderate, and high heterogeneity, respectively [23]. Subgroup meta-analysis was used to analyze sources of heterogeneity. A sensitivity test was conducted using the leave-one-out analysis and the exclusion of studies with few participants. For all tests, *p*-value of < 0.001 was considered to be statistically significant.

## 3. Results

### 3.1. Study Selection

The study selection process for this study is presented in Figure 1. Briefly, our search of four electronic databases returned 4870 records. After removing duplicates and studies satisfying our exclusion criteria, the full texts of 209 studies were evaluated for eligibility. Finally, 95 studies were considered fully eligible and were included in the qualitative and quantitative analyses.

### 3.2. Characteristics of the Eligible Studies

The studies included in this meta-analysis were principally retrospective cohort studies of DM patients at hospital settings, with study periods ranging from 2 to 47 years. Female patients constituted the majority of the enrolled participants, and many of the studies were from the Asian region. Detailed characteristics of the eligible studies are presented in Table 1.

### 3.3. Prevalence of NPC in Dermatomyositis

**Figure 2:**
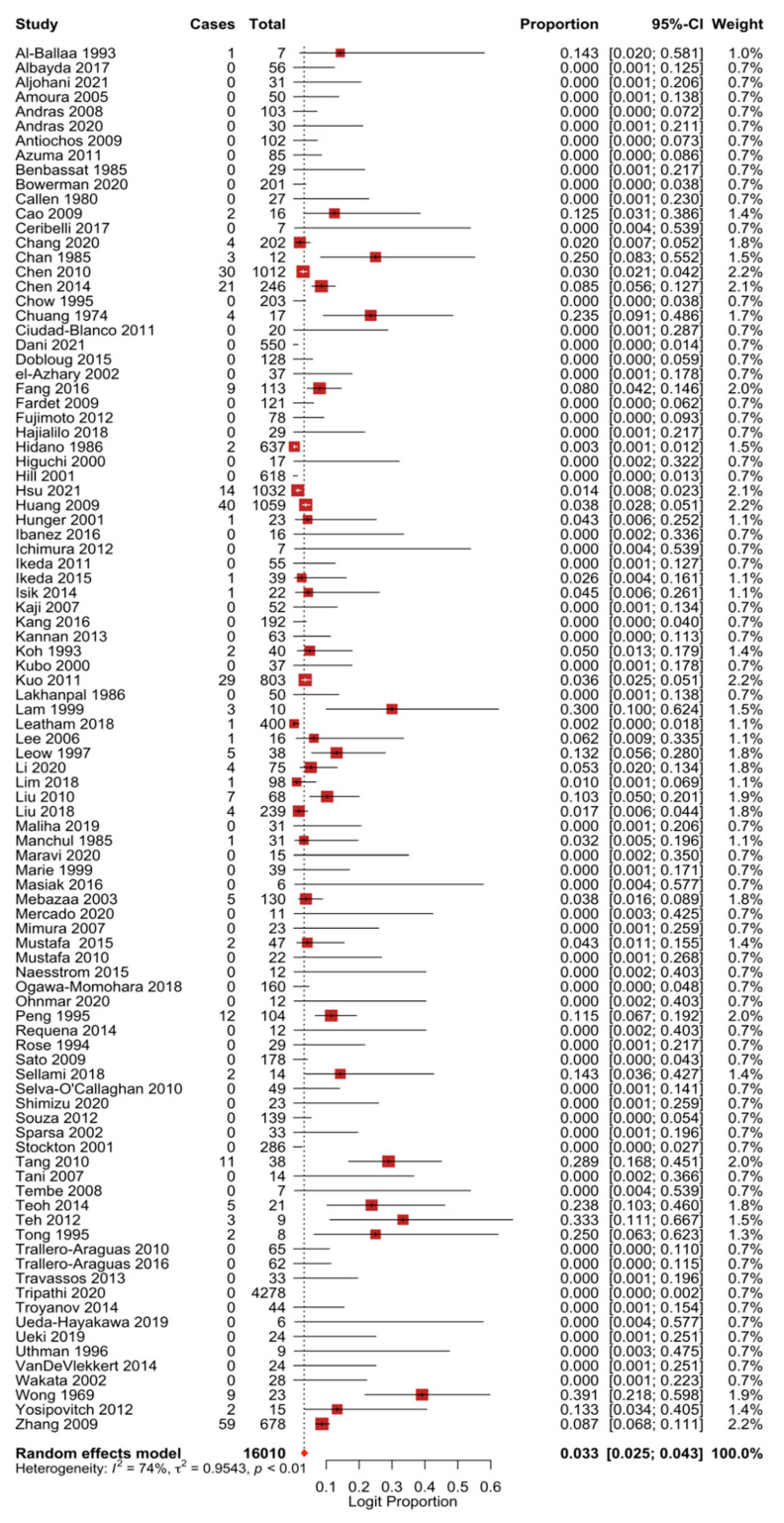
Forest plot of the pooled prevalence of NPC in dermatomyositis patients.

The 95 studies included in this meta-analysis involved a total of 303 NPC cases among 16,010 DM patients. Using the random-effect model, the pooled prevalence of NPC in DM patients was estimated at 3.3% (95% CI, 2.5–4.3) (Figure 2). A relatively high heterogeneity was observed from the statistics (*I*^2^ = 74.03%; Q = 361.951; *p* < 0.001).

### 3.4. Prevalence of NPC in Dermatomyositis Stratified by Study Location and Region

A subgroup meta-analysis was conducted to assess the prevalence of NPC in DM patients from different locations and regions of the globe. From the included studies, data were available for thirty-three locations with the majority of studies from Japan (*n* =16) (Table 2; Figure S1A).

The highest pooled prevalence estimate of 36.5% (95% CI, 22.0–53.9) was observed for Hong Kong, while the least estimate of 0.1% (95% CI, 0.0–1.4) was in Sweden (Table 2; Figure S1A). Studies from India and Taiwan demonstrated a high heterogeneity (*I^2^* = 90.7% and *I^2^* = 86.61%, respectively; *p* < 0.001) and may have contributed to the overall heterogeneity observed.

At the regional level, Asia was the most represented (46 studies) (Table 2; Figure S1B). The highest pooled prevalence estimate of 6.7% (95% CI, 1.8–22.1) was obtained for Africa, while the lowest prevalence of NPC in DM was in North America (0.7%; 95% CI, 0.3–1.8). Studies from Asia demonstrated a high heterogeneity (82.01%).

A higher pooled prevalence (5.2%) was observed in studies whose duration was ten years or below (Table 2; Figure S1C).

### 3.5. Analyses of Sensitivity and Publication Bias

First, the impact of small sample size was evaluated. Ten studies [24,36,56,66,77,98,100,101,107,109] with sample sizes of 10 and below were excluded and the prevalence re-estimated. The resulting prevalence estimate was 2.8% (95% CI, 2.1–3.7; *I*^2^ = 75.03; Q = 336.390; *p* < 0.001) (Figure S2A), indicating a slight decrease from the original prevalence of 3.3%. Sensitivity was further assessed by removing one study at a time (i.e., leave-one-out analysis) using the random-effects model. A prevalence estimate of 3.1% (95% CI, 2.4–4.2) was obtained when the study of Zhang 2009 [113] was removed. This represented the lowest estimate observed following the analysis (Figure S2B). On the other hand, the highest prevalence estimate of 3.5% (95% CI, 2.6–4.5) was observed when the study of Tripathi 2020 [105] was removed. Overall, the NPC prevalence estimates were stable (Figure S2B).

**Figure 3:**
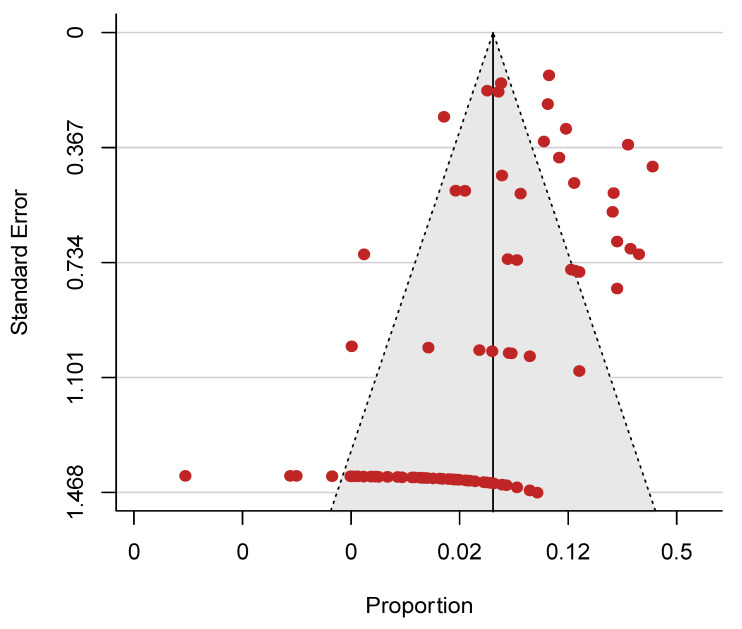
Funnel plot showing evidence of publication bias (Egger’s test, *p* = 0.0008).

The included studies were of good methodological quality (Table S1). A funnel plot was generated for all the included studies (Figure 3). A visual observation of the plot showed a relatively symmetrical plot with evidence of publication bias. In addition, Egger’s regression test for funnel plot asymmetry revealed a significant *p*-value (*p* = 0.0008).

## 4. Discussion

Dermatomyositis (DM) has been associated with underlying malignancies for over a century. The association between DM and cancer was first suggested in 1916 by Stertz [114]. A wide range of malignancies including but not limited to NPC, adenocarcinoma of the lung, breast, pancreas, stomach, colon, and ovary have been described in relation to DM [68,115]. An underlying malignancy is believed to occur in about 15–24% of DM cases [15,115]. Although NPC is considered one of the most common malignancies associated with DM [39,116], the true prevalence of NPC in DM remains unknown as reported prevalence vary with studies. In this systematic review and meta-analysis, an attempt was made to harmonize the various studies reporting the occurrence of NPC in DM patients with the view to deriving a reliable prevalence estimate.

From a total of 2331 relevant articles screened, 95 eligible studies were analyzed. It is noteworthy that over 100 case reports presenting cases of patients with NPC and DM were identified in the course of this review. However, they were excluded since they do not meet the inclusion criteria of a prevalence study. The myriad of case reports encountered encompassed virtually all the regions of the globe. For example, in Europe, Botsios et al. [117] reported a clinical case of a Caucasian Italian patient. Further, in the United Kingdom, Mitra et al. [118] reported the first case of NPC in association with DM. Other similar cases included reports by Boussen et al., Ezejiofor et al. and Boussetta et al. [119,120,121,122] (Africa); Patel et al. [123] (North America); Bica et al. [124] (South America); Low et al. and Kabuto et al. [125,126] (Asia); and Vardi et al. [127] (Oceania). These are indications of the global existence of NPC in DM despite its rarity.

In this study, the prevalence of NPC in DM patients was found to be 3.3% with national estimates hovering between 0.1% and 36.5%. There was a preponderance of studies from the Asian region; about 50% of the studies that contributed to the pooled prevalence estimate obtained were from the region. This could be linked to the fact that NPC is a disease prominent among the Asian population, particularly in Southeast Asia and Southern China [6]. The association of NPC and DM is not commonly reported in the Western population [128]. For instance, despite the identification of some malignancies, NPC was not reported in a pooled analysis of DM cases derived from published national data from Finland, Denmark, and Sweden [52]. Similarly, NPC was not identified in a 21-year retrospective study of a Hungarian DM cohort [28]. The most common malignancies associated with DM in Western country cohorts appear to be lung, breast, ovarian and colorectal cancers [30,52,95].

A close evaluation of the NPC cases analyzed revealed a predominance of male patients (Table 1). Although data on gender were unavailable for the majority of the NPC cases identified, the observed high prevalence of NPC among male DM patients highlights the potential role of gender in the disease condition. Previous studies have established male sex as a predictive factor associated with increased risk of malignancy in dermatomyositis/polymyositis patients [116]. This is in addition to other factors such as cutaneous vasculitis [54,129], cutaneous necrosis [47,130], dysphagia [31,131,132] and periungual erythema [47], among others. The ratio of males to females in the enrolled participants across the studies assessed (Table 1) did not seem to impact the low prevalence of NPC recorded in females, as a relatively high number of participants enrolled in many of the studies were females. Other population-based studies have found ovarian cancer as the most commonly associated malignancy in female DM patients [40,52,133].

Another crucial factor examined in this study was age. We found the majority of the enrolled participants to be adults, and mainly above 40 years, suggesting the predominance of NPC in adult DM. This was, however, expected, as older age had been recognized in many previous studies as a risk factor for malignancy in DM [76,134,135]. Juvenile DM, a childhood-type DM, is extremely uncommon. It affects only about two to four children per million [136]. The association of juvenile DM and malignancy is not well-established, and routine malignancy screening is seldom performed in affected individuals [15]. This perhaps warranted the exclusion of this group from the design of some studies. Nevertheless, cases of juvenile DM were not entirely absent in this study. For example, Huang et al. [14] reported two cases of malignancy-associated DM out of 147 cases involving DM patients under 18 years. Although the comorbid malignancies were reticuloendothelial malignancies, it is noteworthy that both patients were females.

Given that NPC can occur before, after or concurrently with DM diagnosis, we attempted to evaluate the frequency of NPC diagnosis in relation to DM diagnosis among the included studies. Although this detail was inadequately reported (Table 1), studies for which data were available suggest that NPC occurs mostly after the diagnosis of DM, with the diagnosis of NPC ranging from a few weeks to several years after DM diagnosis. In the study conducted by Chen et al. [15], the highest risk of malignancy was found to be within the first year after DM diagnosis. A more detailed study is, however, required to support this assertion. Overall, it is pertinent to prioritize screening for malignancy in DM patients, and particularly NPC among DM patients in the Asian population.

This study has a number of strengths. A major strength is that, to our knowledge, it is the first systematic review and meta-analysis on the prevalence of NPC in DM. Further, a comprehensive search strategy was employed, and a large number of records were screened. In addition, the prevalence estimate obtained was quite stable as confirmed by the sensitivity tests conducted. Lastly, we believe that there is high confidence in the results obtained, since the included studies were of good methodological quality. However, this work is not without limitations, all of which are related to the status of the literature assessed. First, some of the studies included in the analyses were of small sample sizes. Furthermore, information regarding sex, age and period of NPC diagnosis, which are crucial to a comprehensive appraisal of the NPC cases identified, were not reported in some of the studies analyzed in this work.

## 5. Conclusions

In this systematic review and meta-analysis, which to our knowledge is the first report, the prevalence of NPC in DM patients was investigated. A pooled prevalence of 3.3% was derived from published studies from various parts of the world. Based on the findings from this study, screening for NPC in DM patients is highly recommended, particularly among older male DM patients in the Asian region.

## Figures and Tables

**Table 1:** Major characteristics of the included studies reporting the occurrence of NPC in dermatomyositis patients.

Study ID	Study Design	Study Period	Location	Participants			NPC Cases Identified			No. of NPC Diagnosed Relative to DM Diagnosis		
				Total	Age ^1^	Female	Cases	Age ^1^	Female	Before	Concomitant	After
Al-Ballaa 1993 [24]	Retrospective	–	Saudi Arabia	7	–	–	1	35	1	–	–	–
Albayda 2017 [25]	Retrospective	2002–2015	USA	56	–	–	0	–	–	–	–	–
Aljohani 2021 [26]	Retrospective	2017–2018	Saudi Arabia	31	–	–	0	–	–	–	–	–
Amoura 2005 [27]	Retrospective	–	France	50	–	31	0	–	–	–	–	–
Andras 2008 [28]	Retrospective	1985–2006	Hungary	103	43.25 ± 12.6	–	0	–	–	–	–	–
Andras 2020 [29]	Retrospective	1990–2018	Hungary	30	55.9 ± 13.71	21	0	–	–	–	–	–
Antiochos 2009 [30]	Retrospective	1985–2008	USA	102	–	–	0	–	–	–	–	–
Azuma 2011 [31]	Retrospective	1984–2002	Japan	85	–	43	0	–	–	–	–	–
Benbassat 1985 [32]	Retrospective	1956–1976	Israel	29	–	–	0	–	–	–	–	–
Bowerman 2020 [33]	Retrospective	2008–2018	USA	201	51 (39–59)	175	0	–	–	–	–	–
Callen 1980 [34]	Retrospective	1956–1975	USA	27	48.5 ± 13.9	15	0	–	–	–	–	–
Cao 2009 [35]	Retrospective	1998–2004	China	16	50.25 ± NR	10	2	50 and 59	0	–	–	–
Ceribelli 2017 [36]	Retrospective	2013–2016	Italy	7	21–72	5	0	–	–	–	–	–
Chang 2020 [37]	Retrospective	2010–2017	China	202	–	93	4	–	2	–	–	–
Chan 1985 [38]	–	–	Singapore	12	56.7 ± NR	5	3	44.3 ± NR	2	2	1	0
Chen 2010 [39]	Retrospective	1997–2007	Taiwan	1012	41.79 ± 18.96	697	30	–	–	0	0	30
Chen 2014 [15]	Retrospective cohort	2003–2012	China	246	–	139	21	–	2	–	–	–
Chow 1995 [40]	Retrospective	1977–1989	Denmark	203	48.8 ± NR	–	0	–	–	–	–	–
Chuang 1974 [41]	Retrospective	1963–1973	Taiwan	17	44 ± NR	9	4	> 40	0	–	–	–
Ciudad-Blanco 2011 [42]	Retrospective	2007–2010	Spain	20	61 ± NR	19	0	–	–	–	–	–
Dani 2021 [43]	Retrospective	2002–2016	Sweden	550	–	–	0	–	–	–	–	–
Dobloug 2015 [44]	Retrospective	2003–2012	Norway	128	51.4 ± 1.4	87	0	–	–	–	–	–
el-Azhary 2002 [45]	Retrospective	1976–1994	USA	37	–	28	0	–	–	–	–	–
Fang 2016 [46]	Retrospective	2000–2013	Taiwan	113	–	–	9	–	3	0	0	9
Fardet 2009 [47]	Retrospective	1995–2007	France	121	52 (19–77)	85	0	–	–	–	–	–
Fujimoto 2012 [48]	Cohort	2003–2010	Japan	78	–	–	0	–	–	–	–	–
Hajialilo 2018 [49]	Prospective	2004–2016	Iran	29	–	–	0	–	–	–	–	–
Hidano 1986 [50]	Prospective	1973–1983	Japan	637	–	411	2	–	–	–	–	–
Higuchi 2000 [51]	Cohort	1982–1996	Japan	17	–	7	0	–	–	–	–	–
Hill 2001 [52]	Retrospective	1964–1989	Sweden, Denmark, Finland	618	–	–	0	–	–	–	–	–
Hsu 2021 [53]	Retrospective	2001–2019	Taiwan	1032	–	–	14	–	5	4	0	10
Huang 2009 [14]	Retrospective	2000–2005	Taiwan	1059	42.4 ± 19.0	730	40	–	14	10	0	30
Hunger 2001 [54]	Retrospective	1991–1998	Switzerland	23	48 ± NR	14	1	66	0	0	0	1
Ibanez 2016 [55]	Retrospective	2010–2015	Argentina	16	33–76	12	0	–	–	–	–	–
Ichimura 2012 [56]	Retrospective	2003–2010	Japan	7	23–68	1	0	–	–	–	–	–
Ikeda 2011 [57]	Cohort	2000–2009	Japan	55	55.1 ± 14.2	39	0	–	–	–	–	–
Ikeda 2015 [58]	Retrospective	2005–2012	Japan	39	–	28	1	–	–	0	0	1
Isik 2014 [59]	Retrospective	2000–2011	Ankara	22	–	–	1	–	–	–	–	–
Kaji 2007 [60]	Cohort	–	Japan	52	55 ± NR	12	0	–	–	–	–	–
Kang 2016 [61]	Retrospective	–	South Korea	192	–	–	0	–	–	–	–	–
Kannan 2013 [62]	Retrospective	2000–2009	India	63	–	40	0	–	–	–	–	–
Koh 1993 [63]	Retrospective	1986–1991	Singapore	40	52.3 ± NR	22	2	–	0	–	–	–
Kubo 2000 [64]	Cohort	–	Japan	37	–	–	0	–	–	–	–	–
Kuo 2011 [16]	Retrospective	2003–2007	Taiwan	803	44.0 ± 18.3	555	29	–	10	–	–	–
Lakhanpal 1986 [65]	Retrospective	1965–1974	USA	50	–	41	0	–	–	–	–	–
Lam 1999 [66]	Prospective cohort	1988–1996	Hong Kong	10	–	2	3	–	–	–	–	–
Leatham 2018 [67]	Retrospective	1983–2013	USA	400	51.9 (8–84)	323	1	–	–	0	0	1
Lee 2006 [68]	Retrospective	1995–2003	South Korea	16	–	12	1	51	0	0	0	1
Leow 1997 [17]	Retrospective	1989–1994	Singapore	38	53.6 ± NR	21	5	>41	–	2	1	1
Li 2020 [69]	Cohort	2010–2015	China	75	52.89 ± 10.14	39	4	30–61	2	0	0	4
Lim 2018 [70]	Retrospective	1998–2014	Taiwan	98	–	–	1	55	0	–	–	1
Liu 2010 [71]	Retrospective cohort	1996–2006	Singapore	68	50 ± NR	47	7	49.5 ± NR	3	1	3	3
Liu 2018 [72]	Retrospective	1997–2016	China	239	58 (53.0–67.75)	161	4	–	1	–	–	–
Maliha 2019 [73]	Cohort	2005–2018	Canada	31	54 ± 17	24	0	–	–	–	–	–
Manchul 1985 [74]	Retrospective	1965–1980	Canada	31	–	–	1	58	1	0	1	0
Maravi 2020 [75]	Retrospective	2016–2017	Chile	15	53 ± NR	12	0	–	–	–	–	–
Marie 1999 [76]	Retrospective	1983–1997	France	39	–	–	0	–	–	–	–	–
Masiak 2016 [77]	Retrospective	2014–2016	Poland	6	58.7 ± NR	4	0	–	–	–	–	–
Mebazaa 2003 [78]	Retrospective	1982–2000	Tunisia	130	49.6 ± NR	–	5	16–65	2	0	2	3
Mercado 2020 [79]	Prospective	2010–2018	Mexico	11	41.27 ± NR	8	0	–	–	–	–	–
Mimura 2007 [80]	Cohort	–	Japan	23	–	–	0	–	–	–	–	–
Mustafa 2015 [81]	Prospective and retrospective cohort	1996–2014	Jordan	47	36.54 ± 15.61	–	2	51 and 59	0	0	2	0
Mustafa 2010 [82]	Retrospective	1996–2009	Jordan	22	–	–	0	–	–	–	–	–
Naesstrom 2015 [83]	Retrospective	1996–2011	Poland	12	48 ± NR	12	0	–	–	–	–	–
Ogawa-Momohara 2018 [84]	Retrospective	2003–2016	Japan	160	58.7 ± 14.4	110	0	–	–	–	–	–
Ohnmar 2020 [85]	Retrospective	2017–2019	Myanmar	12	46 ± 20	11	0	–	–	–	–	–
Peng 1995 [86]	Retrospective	1970–1993	Taiwan	104	–	–	12	42.8 ± NR	2	2	1	9
Requena 2014 [87]	Retrospective	1994–2013	Spain	12	61 ± NR	6	0	–	–	–	–	–
Rose 1994 [88]	Retrospective	1985–1993	France	29	–	–	0	–	–	–	–	–
Sato 2009 [89]	Retrospective	1986–2007	Brazil	178	–	–	0	–	–	–	–	–
Sellami 2018 [90]	Retrospective	1996–2015	Tunisia	14	57.2 ± NR	10	2	59 and 68	1	1	0	1
Selva-O’Callaghan 2010 [91]	Cohort	2006–2009	Spain	49	–	–	0	–	–	–	–	–
Shimizu 2020 [92]	Cohort	2005–2018	Japan	23	61 ± 14	16	0	–	–	–	–	–
Souza 2012 [93]	Retrospective	1991–2011	Brazil	139	41.7 ± 14.1	111	0	–	–	–	–	–
Sparsa 2002 [94]	Retrospective	1981–2000	France	33	–	–	0	–	–	–	–	–
Stockton 2001 [95]	Retrospective	1982–1996	United Kingdom	286	–	189	0	–	–	–	–	–
Tang 2010 [96]	Retrospective	1997–2009	Malaysia	38	45.7 ± NR	–	11	32–65	3	–	–	–
Tani 2007 [97]	Retrospective	2000–2006	Japan	14	54 ± 14	10	0	–	–	–	–	–
Tembe 2008 [98]	Retrospective	2002–2007	India	7	57 ± NR	7	0	–	–	–	–	–
Teoh 2014 [99]	Retrospective	2000–2010	Malaysia	21	43.8 ± NR	11	5	55.2 ± NR	1	0	3	2
Teh 2012 [100]	Cohort	2006–2009	Malaysia	9	–	–	3	53–56	0	1	1	1
Tong 1995 [101]	Retrospective	1989–1993	Malaysia	8	4–78	6	2	35 and 44	1	1	0	1
Trallero-Araguas 2010 [102]	Cohort	1983–2007	Spain	65	42–69	51	0	–	–	–	–	–
Trallero-Araguas 2016 [103]	Retrospective	–	Spain	62	–	–	0	–	–	–	–	–
Travassos 2013 [104]	Retrospective	1965–2011	Portugal	33	56 ± NR	19	0	–	–	–	–	–
Tripathi 2020 [105]	Retrospective	2009–2015	USA	4278	–	3042	0	–	–	–	–	–
Troyanov 2014 [106]	Prospective	1967–2001	France	44	–	–	0	–	–	–	–	–
Ueda-Hayakawa 2019 [107]	Cohort	–	Japan	6	54 ± NR	5	0	–	–	–	–	–
Ueki 2019 [108]	Retrospective	1990–2016	Japan	24	–	–	0	–	–	–	–	–
Uthman 1996 [109]	Retrospective	1980–1992	France	9	40.2 ± NR	5	0	–	–	–	–	–
VanDeVlekkert 2014 [110]	Prospective	–	The Netherlands	24	–	–	0	–	–	–	–	–
Wakata 2002 [111]	Retrospective	1969–1999	Japan	28	19–74	22	0	–	–	–	–	–
Wong 1969 [12]	Cohort	1963–1968	Hong Kong	23	21–70	8	9	–	–	–	–	–
Yosipovitch 2012 [112]	Prospective cohort	2003–2006	Singapore	15	50 ± 17	10	2	–	–	0	0	2
Zhang 2009 [113]	Retrospective	1974–2008	China	678	–	423	59	–	–	–	–	–

^1^ Age is presented in years [(mean ± SD/median(range/IQR)/range)]; No., number; NPC, nasopharyngeal carcinoma; DM, dermatomyositis; NR, not reported.

**Table 2:** Subgroup analysis. Prevalence of NPC in dermatomyositis patients stratified by study location, geographical region and period of study.

Subgroup	No of Studies	Prevalence (%)	95% CI	*I*^2^ (%)	Q	Heterogeneity Test	
						DF	*p*
Location							
Saudi Arabia	2	5.8	0.6–37.6	42.15	1.729	1	0.189
USA	8	0.4	0.1–1.1	23.45	9.144	7	0.242
France	7	1.3	0.5–3.7	0	1.678	6	0.947
Hungary	2	0.9	0.1–6.0	0	0.369	1	0.543
Japan	16	1.2	0.6–2.2	0	10.017	15	0.819
Israel	1	1.7	0.1–21.7	–	–	–	–
China	6	5.4	3.1–9.1	75.01	20.004	5	0.001
Italy	1	6.3	0.4–53.9	–	–	–	–
Singapore	5	11.9	7.7–17.9	0	3.777	4	0.437
Taiwan	8	4.6	2.8–7.4	86.61	52.293	7	<0.001
Denmark	1	0.2	0.0–3.8	–	–	–	–
Spain	5	1.4	0.4–4.7	0	1.070	4	0.899
Sweden	1	0.1	0.0–1.4	–	–	–	–
Norway	1	0.4	0.0–5.9	–	–	–	–
Iran	1	1.7	0.1–21.7	–	–	–	–
Switzerland	1	4.3	0.6–25.2	–	–	–	–
Argentina	1	2.9	0.2–33.6	–	–	–	–
Ankara	1	4.5	0.6–26.1	–	–	–	–
South Korea	2	1.5	0.1–26.5	70.83	3.428	1	0.064
India	2	2.2	0.3–15.3	90.7	1.100	1	0.294
Hong Kong	2	36.5	22.0–53.9	0	0.250	1	0.617
Canada	2	2.5	0.5–11.6	0	0.180	1	0.672
Chile	1	3.1	0.2–35.0	–	–	–	–
Poland	2	5.2	0.7–29.3	0	0.101	1	0.751
Tunisia	2	6.7	1.8–22.1	61.15	2.574	1	0.109
Mexico	1	4.2	0.3–42.5	–	–	–	–
Jordan	2	3.7	1.1–12.0	0	0.187	1	0.665
Myanmar	1	3.8	0.2–40.3	–	–	–	–
Brazil	2	0.3	0.0–2.2	0	0.015	1	0.902
United Kingdom	1	0.2	0.0–2.7	–	–	–	–
Malaysia	4	27.7	18.8–38.9	0	0.358	3	0.949
Portugal	1	1.5	0.1–19.6	–	–	–	–
The Netherlands	1	2.0	0.1–25.1	–	–	–	–
Overall	94	3.4	2.6–4.5	73.66	353.089	93	<0.001
**Region**							
Middle East	6	3.7	1.5–8.7	0	3.076	5	0.688
North America	11	0.7	0.3–1.8	33.56	15.051	10	0.130
Europe	25	1.1	0.7–1.9	0	19.525	24	0.723
Asia	47	5.7	4.1–7.9	81.61	250.180	46	<0.001
South America	4	1.0	0.2–3.8	0	2.592	3	0.459
Africa	2	6.7	1.8–22.1	61.15	2.574	1	0.109
Overall	95	3.3	2.5–4.3	74.03	361.951	94	<0.001
**Study period**							
More than 10 years	50	2.3	1.5–3.4	78.28	225.627	49	<0.001
Ten years or less	35	5.2	3.4–7.8	70.75	116.256	34	<0.001
Overall	85	3.3	2.5–4.4	75.45	342.171	84	<0.001

## Data Availability

All data accessed and analyzed in this study are available in the article and its Supplementary Materials.

## Supplementary Materials

The following are available online at https://www.mdpi.com/article/10.3390/cancers13081886/s1, Figure S1: Forest plot of the pooled prevalence of NPC in dermatomyositis patients stratified by study location, geographical region and period of study, Figure S2: Sensitivity tests, Table S1: Quality assessment of included studies, File S1: Search strategy, File S2: Joanna Briggs Institute (JBI) critical appraisal checklist for prevalence studies.
