# Supplementary material for: Prevalence of Nasopharyngeal Carcinoma in Patients with Dermatomyositis: A Systematic Review and Meta-Analysis
Source: Cancers (Basel). 2021 Apr 14;13(8):1886. doi: 10.3390/cancers13081886 (PMC8071042; doi:10.3390/cancers13081886)
Supplement: Supplementary file 1 [file cancers-13-01886-s001.zip › Figure S1.pdf]

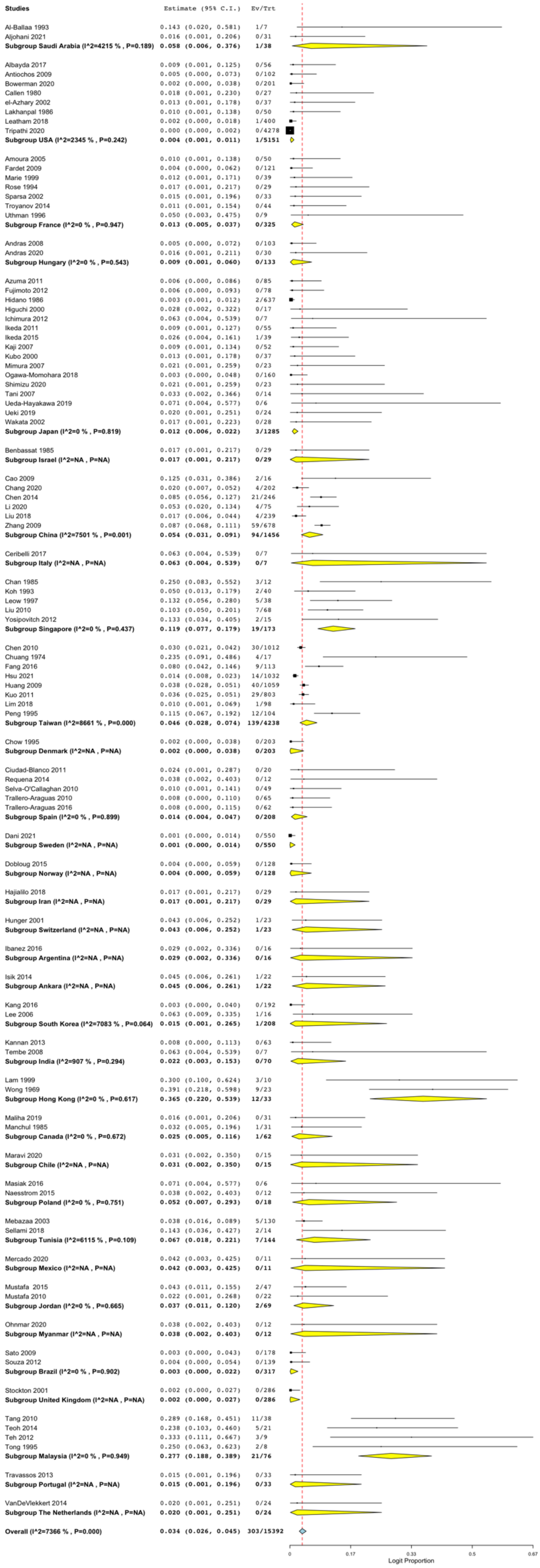

Figure S1-A. Forest plot of the pooled prevalence of NPC in dermatomyositis patients stratified by study location

Studies	Estimate (95% C.I.)	Ev/Trt
Al-Ballaa 1993	0.143 (0.020, 0.581)	1/7
Aljohani 2021	0.016 (0.001, 0.206)	0/31
Benbassat 1985	0.017 (0.001, 0.217)	0/29
Hajialilo 2018	0.017 (0.001, 0.217)	0/29
Mustafa 2015	0.043 (0.011, 0.155)	2/47
Mustafa 2010	0.022 (0.001, 0.268)	0/22
**Subgroup Middle East (I^2=0 % , P=0.688)**	**0.037 (0.015, 0.087)**	**3/165**
Albayda 2017	0.009 (0.001, 0.125)	0/56
Antiochos 2009	0.005 (0.000, 0.073)	0/102
Bowerman 2020	0.002 (0.000, 0.038)	0/201
Callen 1980	0.018 (0.001, 0.230)	0/27
el-Azhary 2002	0.013 (0.001, 0.178)	0/37
Lakhanpal 1986	0.010 (0.001, 0.138)	0/50
Leatham 2018	0.002 (0.000, 0.018)	1/400
Maliha 2019	0.016 (0.001, 0.206)	0/31
Manchul 1985	0.032 (0.005, 0.196)	1/31
Mercado 2020	0.042 (0.003, 0.425)	0/11
Tripathi 2020	0.000 (0.000, 0.002)	0/4278
**Subgroup North America (I^2=3356 % , P=0.130)**	**0.007 (0.003, 0.018)**	**2/5224**
Amoura 2005	0.010 (0.001, 0.138)	0/50
Andras 2008	0.005 (0.000, 0.072)	0/103
Andras 2020	0.016 (0.001, 0.211)	0/30
Ceribelli 2017	0.063 (0.004, 0.539)	0/7
Chow 1995	0.002 (0.000, 0.038)	0/203
Ciudad-Blanco 2011	0.024 (0.001, 0.287)	0/20
Dani 2021	0.001 (0.000, 0.014)	0/550
Dobloug 2015	0.004 (0.000, 0.059)	0/128
Fardet 2009	0.004 (0.000, 0.062)	0/121
Hill 2001	0.001 (0.000, 0.013)	0/618
Hunger 2001	0.043 (0.006, 0.252)	1/23
Marie 1999	0.012 (0.001, 0.171)	0/39
Masiak 2016	0.071 (0.004, 0.577)	0/6
Naesstrom 2015	0.038 (0.002, 0.403)	0/12
Requena 2014	0.038 (0.002, 0.403)	0/12
Rose 1994	0.017 (0.001, 0.217)	0/29
Selva-O'Callaghan 2010	0.010 (0.001, 0.141)	0/49
Sparsa 2002	0.015 (0.001, 0.196)	0/33
Stockton 2001	0.002 (0.000, 0.027)	0/286
Trallero-Araguas 2010	0.008 (0.000, 0.110)	0/65
Trallero-Araguas 2016	0.008 (0.000, 0.115)	0/62
Travassos 2013	0.015 (0.001, 0.196)	0/33
Troyanov 2014	0.011 (0.001, 0.154)	0/44
Uthman 1996	0.050 (0.003, 0.475)	0/9
VanDeVlekkert 2014	0.020 (0.001, 0.251)	0/24
**Subgroup Europe (I^2=0 % , P=0.723)**	**0.011 (0.007, 0.019)**	**1/2556**
Azuma 2011	0.006 (0.000, 0.086)	0/85
Cao 2009	0.125 (0.031, 0.386)	2/16
Chang 2020	0.020 (0.007, 0.052)	4/202
Chan 1985	0.250 (0.083, 0.552)	3/12
Chen 2010	0.030 (0.021, 0.042)	30/1012
Chen 2014	0.085 (0.056, 0.127)	21/246
Chuang 1974	0.235 (0.091, 0.486)	4/17
Fang 2016	0.080 (0.042, 0.146)	9/113
Fujimoto 2012	0.006 (0.000, 0.093)	0/78
Hidano 1986	0.003 (0.001, 0.012)	2/637
Higuchi 2000	0.028 (0.002, 0.322)	0/17
Hsu 2021	0.014 (0.008, 0.023)	14/1032
Huang 2009	0.038 (0.028, 0.051)	40/1059
Ichimura 2012	0.063 (0.004, 0.539)	0/7
Ikeda 2011	0.009 (0.001, 0.127)	0/55
Ikeda 2015	0.026 (0.004, 0.161)	1/39
Isik 2014	0.045 (0.006, 0.261)	1/22
Kaji 2007	0.009 (0.001, 0.134)	0/52
Kang 2016	0.003 (0.000, 0.040)	0/192
Kannan 2013	0.008 (0.000, 0.113)	0/63
Koh 1993	0.050 (0.013, 0.179)	2/40
Kubo 2000	0.013 (0.001, 0.178)	0/37
Kuo 2011	0.036 (0.025, 0.051)	29/803
Lam 1999	0.300 (0.100, 0.624)	3/10
Lee 2006	0.063 (0.009, 0.335)	1/16
Leow 1997	0.132 (0.056, 0.280)	5/38
Li 2020	0.053 (0.020, 0.134)	4/75
Lim 2018	0.010 (0.001, 0.069)	1/98
Liu 2010	0.103 (0.050, 0.201)	7/68
Liu 2018	0.017 (0.006, 0.044)	4/239
Mimura 2007	0.021 (0.001, 0.259)	0/23
Ogawa-Momohara 2018	0.003 (0.000, 0.048)	0/160
Ohnmar 2020	0.038 (0.002, 0.403)	0/12
Peng 1995	0.115 (0.067, 0.192)	12/104
Shimizu 2020	0.021 (0.001, 0.259)	0/23
Tang 2010	0.289 (0.168, 0.451)	11/38
Tani 2007	0.033 (0.002, 0.366)	0/14
Tembe 2008	0.063 (0.004, 0.539)	0/7
Teoh 2014	0.238 (0.103, 0.460)	5/21
Teh 2012	0.333 (0.111, 0.667)	3/9
Tong 1995	0.250 (0.063, 0.623)	2/8
Ueda-Hayakawa 2019	0.071 (0.004, 0.577)	0/6
Ueki 2019	0.020 (0.001, 0.251)	0/24
Wakata 2002	0.017 (0.001, 0.223)	0/28
Wong 1969	0.391 (0.218, 0.598)	9/23
Yosipovitch 2012	0.133 (0.034, 0.405)	2/15
Zhang 2009	0.087 (0.068, 0.111)	59/678
**Subgroup Asia (I^2=8161 % , P=0.000)**	**0.057 (0.041, 0.079)**	**290/7573**
Ibanez 2016	0.029 (0.002, 0.336)	0/16
Maravi 2020	0.031 (0.002, 0.350)	0/15
Sato 2009	0.003 (0.000, 0.043)	0/178
Souza 2012	0.004 (0.000, 0.054)	0/139
**Subgroup South America (I^2=0 % , P=0.459)**	**0.010 (0.002, 0.038)**	**0/348**
Mebazaa 2003	0.038 (0.016, 0.089)	5/130
Sellami 2018	0.143 (0.036, 0.427)	2/14
**Subgroup Africa (I^2=6115 % , P=0.109)**	**0.067 (0.018, 0.221)**	**7/144**
**Overall (I^2=7403 % , P=0.000)**	**0.033 (0.025, 0.043)**	**303/16010**

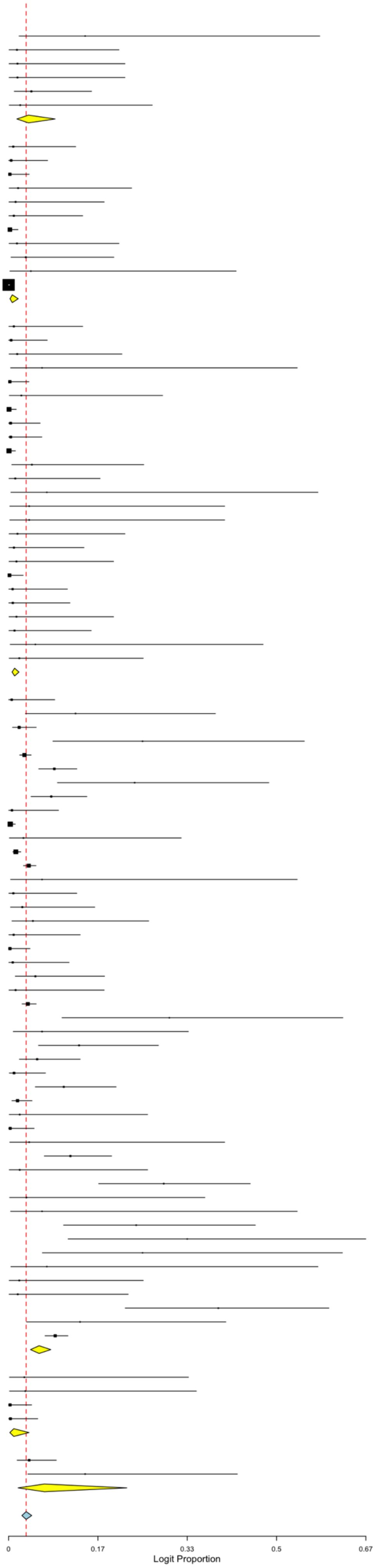

Figure S1-B. Forest plot of the pooled prevalence of NPC in dermatomyositis patients stratified by geographical region

Studies	Estimate (95% C.I.)	Ev/Trt
Albayda 2017	0.009 (0.001, 0.125)	0/56
Andras 2008	0.005 (0.000, 0.072)	0/103
Andras 2020	0.016 (0.001, 0.211)	0/30
Antiochos 2009	0.005 (0.000, 0.073)	0/102
Azuma 2011	0.006 (0.000, 0.086)	0/85
Benbassat 1985	0.017 (0.001, 0.217)	0/29
Bowerman 2020	0.002 (0.000, 0.038)	0/201
Callen 1980	0.018 (0.001, 0.230)	0/27
Chen 2010	0.030 (0.021, 0.042)	30/1012
Chow 1995	0.002 (0.000, 0.038)	0/203
Chuang 1974	0.235 (0.091, 0.486)	4/17
Dani 2021	0.001 (0.000, 0.014)	0/550
el-Azhary 2002	0.013 (0.001, 0.178)	0/37
Fang 2016	0.080 (0.042, 0.146)	9/113
Fardet 2009	0.004 (0.000, 0.062)	0/121
Hajialilo 2018	0.017 (0.001, 0.217)	0/29
Hidano 1986	0.003 (0.001, 0.012)	2/637
Higuchi 2000	0.028 (0.002, 0.322)	0/17
Hill 2001	0.001 (0.000, 0.013)	0/618
Hsu 2021	0.014 (0.008, 0.023)	14/1032
Isik 2014	0.045 (0.006, 0.261)	1/22
Leatham 2018	0.002 (0.000, 0.018)	1/400
Lim 2018	0.010 (0.001, 0.069)	1/98
Liu 2010	0.103 (0.050, 0.201)	7/68
Liu 2018	0.017 (0.006, 0.044)	4/239
Maliha 2019	0.016 (0.001, 0.206)	0/31
Manchul 1985	0.032 (0.005, 0.196)	1/31
Marie 1999	0.012 (0.001, 0.171)	0/39
Mebazaa 2003	0.038 (0.016, 0.089)	5/130
Mustafa 2015	0.043 (0.011, 0.155)	2/47
Mustafa 2010	0.022 (0.001, 0.268)	0/22
Naesstrom 2015	0.038 (0.002, 0.403)	0/12
Ogawa-Momohara 2018	0.003 (0.000, 0.048)	0/160
Peng 1995	0.115 (0.067, 0.192)	12/104
Requena 2014	0.038 (0.002, 0.403)	0/12
Sato 2009	0.003 (0.000, 0.043)	0/178
Sellami 2018	0.143 (0.036, 0.427)	2/14
Shimizu 2020	0.021 (0.001, 0.259)	0/23
Souza 2012	0.004 (0.000, 0.054)	0/139
Sparsa 2002	0.015 (0.001, 0.196)	0/33
Stockton 2001	0.002 (0.000, 0.027)	0/286
Tang 2010	0.289 (0.168, 0.451)	11/38
Teoh 2014	0.238 (0.103, 0.460)	5/21
Trallero-Araguas 2010	0.008 (0.000, 0.110)	0/65
Travassos 2013	0.015 (0.001, 0.196)	0/33
Troyanov 2014	0.011 (0.001, 0.154)	0/44
Ueki 2019	0.020 (0.001, 0.251)	0/24
Uthman 1996	0.050 (0.003, 0.475)	0/9
Wakata 2002	0.017 (0.001, 0.223)	0/28
Zhang 2009	0.087 (0.068, 0.111)	59/678
**Subgroup More than 10 years (I^2=7828 % , P=0.000)**	**0.023 (0.015, 0.034)**	**170/8047**
Aljohani 2021	0.016 (0.001, 0.206)	0/31
Cao 2009	0.125 (0.031, 0.386)	2/16
Ceribelli 2017	0.063 (0.004, 0.539)	0/7
Chang 2020	0.020 (0.007, 0.052)	4/202
Chen 2014	0.085 (0.056, 0.127)	21/246
Ciudad-Blanco 2011	0.024 (0.001, 0.287)	0/20
Dobloug 2015	0.004 (0.000, 0.059)	0/128
Fujimoto 2012	0.006 (0.000, 0.093)	0/78
Huang 2009	0.038 (0.028, 0.051)	40/1059
Hunger 2001	0.043 (0.006, 0.252)	1/23
Ibanez 2016	0.029 (0.002, 0.336)	0/16
Ichimura 2012	0.063 (0.004, 0.539)	0/7
Ikeda 2011	0.009 (0.001, 0.127)	0/55
Ikeda 2015	0.026 (0.004, 0.161)	1/39
Kannan 2013	0.008 (0.000, 0.113)	0/63
Koh 1993	0.050 (0.013, 0.179)	2/40
Kuo 2011	0.036 (0.025, 0.051)	29/803
Lakhanpal 1986	0.010 (0.001, 0.138)	0/50
Lam 1999	0.300 (0.100, 0.624)	3/10
Lee 2006	0.063 (0.009, 0.335)	1/16
Leow 1997	0.132 (0.056, 0.280)	5/38
Li 2020	0.053 (0.020, 0.134)	4/75
Maravi 2020	0.031 (0.002, 0.350)	0/15
Masiak 2016	0.071 (0.004, 0.577)	0/6
Mercado 2020	0.042 (0.003, 0.425)	0/11
Ohnmar 2020	0.038 (0.002, 0.403)	0/12
Rose 1994	0.017 (0.001, 0.217)	0/29
Selva-O'Callaghan 2010	0.010 (0.001, 0.141)	0/49
Tani 2007	0.033 (0.002, 0.366)	0/14
Tembe 2008	0.063 (0.004, 0.539)	0/7
Teh 2012	0.333 (0.111, 0.667)	3/9
Tong 1995	0.250 (0.063, 0.623)	2/8
Tripathi 2020	0.000 (0.000, 0.002)	0/4278
Wong 1969	0.391 (0.218, 0.598)	9/23
Yosipovitch 2012	0.133 (0.034, 0.405)	2/15
**Subgroup Ten years or less (I^2=7075 % , P=0.000)**	**0.052 (0.034, 0.078)**	**129/7498**
**Overall (I^2=7545 % , P=0.000)**	**0.033 (0.025, 0.044)**	**299/15545**

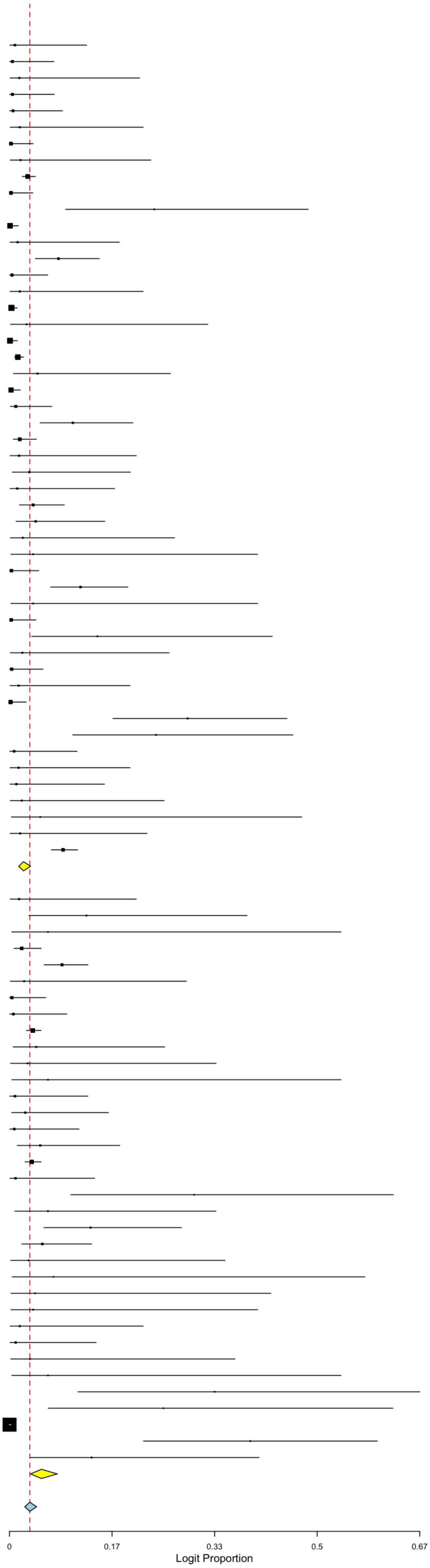

Figure S1-C. Forest plot of the pooled prevalence of NPC in dermatomyositis patients stratified by study period
