# Supplementary material for: Prevalence of Nasopharyngeal Carcinoma in Patients with Dermatomyositis: A Systematic Review and Meta-Analysis
Source: Cancers (Basel). 2021 Apr 14;13(8):1886. doi: 10.3390/cancers13081886 (PMC8071042; doi:10.3390/cancers13081886)
Supplement: Supplementary file 1 [file cancers-13-01886-s001.zip › File S1.pdf]

Search Strategy

Pubmed

(((Dermatomyositis[Title/Abstract]) OR (Dermatopolymyositis[Title/Abstract])) OR (Myositis[Title/Abstract])) AND ((((("Nasopharyngeal carcinoma"[Title/Abstract]) OR ("Nasopharyngeal cancer"[Title/Abstract])) OR ("Nasopharyngeal neoplasm"[Title/Abstract])) OR (NPC[Title/Abstract])) OR (Malignancy[Title/Abstract])) OR (Cancer[Title/Abstract]))

Scopus

TITLE-ABS(Dermatomyositis OR Dermatopolymyositis OR Myositis) AND TITLE-ABS("Nasopharyngeal carcinoma" OR "Nasopharyngeal cancer" OR "Nasopharyngeal neoplasm" OR NPC OR Malignancy OR Cancer)

ScienceDirect

(Dermatomyositis OR Dermatopolymyositis OR Myositis) ("Nasopharyngeal carcinoma" OR "Nasopharyngeal cancer" OR "Nasopharyngeal neoplasm" OR NPC OR Malignancy OR Cancer)

Google Scholar

allintitle:(Dermatomyositis OR Dermatopolymyositis OR Myositis) ("Nasopharyngeal carcinoma" OR "Nasopharyngeal cancer" OR "Nasopharyngeal neoplasm" OR NPC OR Malignancy OR Cancer)
