# Supplementary material for: Prevalence of Nasopharyngeal Carcinoma in Patients with Dermatomyositis: A Systematic Review and Meta-Analysis
Source: Cancers (Basel). 2021 Apr 14;13(8):1886. doi: 10.3390/cancers13081886 (PMC8071042; doi:10.3390/cancers13081886)
Supplement: Supplementary file 1 [file cancers-13-01886-s001.zip › Table S1.pdf]

Quality of included studies by JBI critical appraisal checklist for studies reporting prevalence data

Study ID		Checklist*									Overall
		1	2	3	4	5	6	7	8	9	
1	Al-Ballaa 1993	yes	yes	no	no	yes	yes	yes	yes	yes	7
2	Albayda 2017	yes	yes	yes	yes	yes	yes	yes	yes	yes	9
3	Aljohani 2021	yes	yes	yes	yes	yes	yes	yes	yes	yes	9
4	Amoura 2005	yes	yes	yes	no	yes	yes	yes	yes	yes	8
5	Andras 2008	yes	yes	yes	yes	yes	yes	yes	yes	yes	9
6	Andras 2020	yes	yes	yes	yes	yes	yes	yes	yes	yes	9
7	Antiochos 2009	yes	yes	yes	yes	yes	yes	yes	yes	yes	9
8	Azuma 2011	yes	yes	yes	yes	yes	yes	yes	yes	yes	9
9	Benbassat 1985	yes	yes	yes	yes	yes	yes	yes	yes	yes	9
10	Bowerman 2020	yes	yes	yes	yes	yes	yes	yes	yes	yes	9
11	Callen 1980	yes	yes	yes	yes	yes	yes	yes	yes	yes	9
12	Cao 2009	yes	yes	yes	yes	yes	yes	yes	yes	yes	9
13	Ceribelli 2017	yes	yes	no	yes	yes	yes	yes	yes	yes	8
14	Chang 2020	yes	yes	yes	yes	yes	yes	yes	yes	yes	9
15	Chan 1985	yes	yes	yes	no	yes	yes	yes	yes	yes	8
16	Chen 2010	yes	yes	yes	yes	yes	yes	yes	yes	yes	9
17	Chen 2014	yes	yes	yes	yes	yes	yes	yes	yes	yes	9
18	Chow 1995	yes	yes	yes	yes	yes	yes	yes	yes	yes	9
19	Chuang 1974	yes	yes	yes	yes	yes	yes	yes	yes	yes	9
20	Ciudad-Blanco 2011	yes	yes	yes	yes	yes	yes	yes	yes	yes	9
21	Dani 2021	yes	yes	yes	yes	yes	yes	yes	yes	yes	9
22	Dobloug 2015	yes	yes	yes	yes	yes	yes	yes	yes	yes	9
23	el-Azhary 2002	yes	yes	yes	yes	yes	yes	yes	yes	yes	9
24	Fang 2016	yes	yes	yes	yes	yes	yes	yes	yes	yes	9
25	Fardet 2009	yes	yes	yes	yes	yes	yes	yes	yes	yes	9
26	Fujimoto 2012	yes	yes	yes	yes	yes	yes	yes	yes	yes	9
27	Hajialilo 2018	yes	yes	yes	yes	yes	yes	yes	yes	yes	9
28	Hidano 1986	yes	yes	yes	yes	yes	yes	yes	yes	yes	9
29	Higuchi 2000	yes	yes	yes	yes	yes	yes	yes	yes	yes	9
30	Hill 2001	yes	yes	yes	yes	yes	yes	yes	yes	yes	9
31	Hsu 2021	yes	yes	yes	yes	yes	yes	yes	yes	yes	9
32	Huang 2009	yes	yes	yes	yes	yes	yes	yes	yes	yes	9
33	Hunger 2001	yes	yes	yes	yes	yes	yes	yes	yes	yes	9
34	Ibanez 2016	yes	yes	yes	yes	yes	yes	yes	yes	yes	9
35	Ichimura 2012	yes	yes	no	yes	yes	yes	yes	yes	yes	8
36	Ikeda 2011	yes	yes	yes	yes	yes	yes	yes	yes	yes	9
37	Ikeda 2015	yes	yes	yes	yes	yes	yes	yes	yes	yes	9
38	Isik 2014	yes	yes	yes	yes	yes	yes	yes	yes	yes	9
39	Kaji 2007	yes	yes	yes	no	yes	yes	yes	yes	yes	8
40	Kang 2016	yes	yes	yes	no	yes	yes	yes	yes	yes	8
41	Kannan 2013	yes	yes	yes	yes	yes	yes	yes	yes	yes	9

42	Koh 1993	yes	yes	yes	yes	yes	yes	yes	yes	yes	9
43	Kubo 2000	yes	yes	yes	no	yes	yes	yes	yes	yes	8
44	Kuo 2011	yes	yes	yes	yes	yes	yes	yes	yes	yes	9
45	Lakhanpal 1986	yes	yes	yes	yes	yes	yes	yes	yes	yes	9
46	Lam 1999	yes	yes	no	yes	yes	yes	yes	yes	yes	8
47	Leatham 2018	yes	yes	yes	yes	yes	yes	yes	yes	yes	9
48	Lee 2006	yes	yes	yes	yes	yes	yes	yes	yes	yes	9
49	Leow 1997	yes	yes	yes	yes	yes	yes	yes	yes	yes	9
50	Li 2020	yes	yes	yes	yes	yes	yes	yes	yes	yes	9
51	Lim 2018	yes	yes	yes	yes	yes	yes	yes	yes	yes	9
52	Liu 2010	yes	yes	yes	yes	yes	yes	yes	yes	yes	9
53	Liu 2018	yes	yes	yes	yes	yes	yes	yes	yes	yes	9
54	Maliha 2019	yes	yes	yes	yes	yes	yes	yes	yes	yes	9
55	Manchul 1985	yes	yes	yes	yes	yes	yes	yes	yes	yes	9
56	Maravi 2020	yes	yes	yes	yes	yes	yes	yes	yes	yes	9
57	Marie 1999	yes	yes	yes	yes	yes	yes	yes	yes	yes	9
58	Masiak 2016	yes	yes	no	yes	yes	yes	yes	yes	yes	8
59	Mebazaa 2003	yes	yes	yes	yes	yes	yes	yes	yes	yes	9
60	Mercado 2020	yes	yes	yes	yes	yes	yes	yes	yes	yes	9
61	Mimura 2007	yes	yes	yes	no	yes	yes	yes	yes	yes	8
62	Mustafa 2015	yes	yes	yes	yes	yes	yes	yes	yes	yes	9
63	Mustafa 2010	yes	yes	yes	yes	yes	yes	yes	yes	yes	9
64	Naesstrom 2015	yes	yes	yes	yes	yes	yes	yes	yes	yes	9
65	Ogawa-Momohara 2018	yes	yes	yes	yes	yes	yes	yes	yes	yes	9
66	Ohnmar 2020	yes	yes	yes	yes	yes	yes	yes	yes	yes	9
67	Peng 1995	yes	yes	yes	yes	yes	yes	yes	yes	yes	9
68	Requena 2014	yes	yes	yes	yes	yes	yes	yes	yes	yes	9
69	Rose 1994	yes	yes	yes	yes	yes	yes	yes	yes	yes	9
70	Sato 2009	yes	yes	yes	yes	yes	yes	yes	yes	yes	9
71	Sellami 2018	yes	yes	yes	yes	yes	yes	yes	yes	yes	9
72	Selva-O'Callaghan 2010	yes	yes	yes	yes	yes	yes	yes	yes	yes	9
73	Shimizu 2020	yes	yes	yes	yes	yes	yes	yes	yes	yes	9
74	Souza 2012	yes	yes	yes	yes	yes	yes	yes	yes	yes	9
75	Sparsa 2002	yes	yes	yes	yes	yes	yes	yes	yes	yes	9
76	Stockton 2001	yes	yes	yes	yes	yes	yes	yes	yes	yes	9
77	Tang 2010	yes	yes	yes	yes	yes	yes	yes	yes	yes	9
78	Tani 2007	yes	yes	yes	yes	yes	yes	yes	yes	yes	9
79	Tembe 2008	yes	yes	no	yes	yes	yes	yes	yes	yes	8
80	Teoh 2014	yes	yes	yes	yes	yes	yes	yes	yes	yes	9
81	Teh 2012	yes	yes	no	yes	yes	yes	yes	yes	yes	8
82	Tong 1995	yes	yes	no	yes	yes	yes	yes	yes	yes	8
83	Trallero-Araguas 2010	yes	yes	yes	yes	yes	yes	yes	yes	yes	9
84	Trallero-Araguas 2016	yes	yes	yes	no	yes	yes	yes	yes	yes	8
85	Travassos 2013	yes	yes	yes	yes	yes	yes	yes	yes	yes	9
86	Tripathi 2020	yes	yes	yes	yes	yes	yes	yes	yes	yes	9
87	Troyanov 2014	yes	yes	yes	yes	yes	yes	yes	yes	yes	9

88	Ueda-Hayakawa 2019	yes	yes	no	no	yes	yes	yes	yes	yes	7
89	Ueki 2019	yes	yes	yes	yes	yes	yes	yes	yes	yes	9
90	Uthman 1996	yes	yes	no	yes	yes	yes	yes	yes	yes	8
91	VanDeVlekkert 2014	yes	yes	yes	no	yes	yes	yes	yes	yes	8
92	Wakata 2002	yes	yes	yes	yes	yes	yes	yes	yes	yes	9
93	Wong 1969	yes	yes	yes	yes	yes	yes	yes	yes	yes	9
94	Yosipovitch 2012	yes	yes	yes	yes	yes	yes	yes	yes	yes	9
95	Zhang 2009	yes	yes	yes	yes	yes	yes	yes	yes	yes	9

*** 1.** Appropriate sampling frame to address target population, **2.** Appropriate sampling way of study participants, **3.** Adequate sample size, **4.** Detail description of study participants and settings, **5.** Data analysis with sufficient coverage of identified sample, **6.** Use of valid methods to identify the condition, **7.** Standard, reliable way of measurement of condition for all participants, **8.** Availability of appropriate statistical analysis, **9.** Adequate response rate and management of low response rate.
